# Regulation of the immune microenvironment by SUMO in diabetes mellitus

**DOI:** 10.3389/fimmu.2025.1506500

**Published:** 2025-02-26

**Authors:** Yuting Zhuo, Shangui Fu, Yue Qiu

**Affiliations:** ^1^ Department of Endocrinology and Metabolism, Jiujiang Hospital of Traditional Chinese Medicine, Jiujiang, Jiangxi, China; ^2^ The Second School of Clinical Medicine, Jiangxi Medical College, Nanchang University, Nanchang, Jiangxi, China

**Keywords:** immune, SUMOylation, diabetes mellitus, inflammation, posttranslation modification, signaling pathway

## Abstract

Post-translational modifications such as SUMOylation are crucial for the functionality and signal transduction of a diverse array of proteins. Analogous to ubiquitination, SUMOylation has garnered significant attention from researchers and has been implicated in the pathogenesis of various human diseases in recent years, such as cancer, neurological lesions, cardiovascular diseases, diabetes mellitus, and so on. The pathogenesis of diabetes, particularly type 1 and type 2 diabetes, has been closely associated with immune dysfunction, which constitutes the primary focus of this review. This review will elucidate the process of SUMOylation and its impact on diabetes mellitus development and associated complications, focusing on its regulatory effects on the immune microenvironment. This article summarizes various signaling pathways at both cellular and molecular levels that are implicated in these processes. Furthermore, it proposes potential new targets for drug development aimed at the prevention and treatment of diabetes mellitus based on insights gained from the SUMOylation process.

## 1 Introduction

In the context of globalization, diabetes mellitus (DM) has emerged as a critical public health issue and a substantial economic burden on healthcare systems worldwide. The estimated prevalence of DM in the adult population was 10.5% in 2021, with projections indicating a continued rise in prevalence, as reported in the survey (1). From 2007 to 2017, there was a notable increase in the prevalence of DM among Chinese adults, and the number of diabetes-related risk factors remained uncontrolled (2). Therefore, addressing the challenge posed by DM has emerged as an urgent priority for the public health protection system. DM is a group of metabolic disorders that are characterized by hyperglycemia. Based on the diagnostic and classification criteria set forth by the American Diabetes Association (ADA), DM is classified into four categories: type 1 diabetes mellitus (T1DM), type 2 diabetes mellitus(T2DM), gestational diabetes mellitus (GDM), and other types of DM. T1DM is regarded as an autoimmune disease, which is characterized by the autoimmune destruction of pancreatic β-cells. T2DM constitutes over 90% of all DM types, with a more intricate underlying mechanism than that of T1DM. This condition is marked by insulin resistance and a relative insulin deficiency (3, 4). DM is frequently a multifactorial disease that can present in various forms, including immune dysregulation, glucose metabolism disorders, insulin resistance, and β-cell destruction (5). In these cases, immune dysfunction exerts an influence on the development of DM, which is caused by various factors and contributes to the onset of DM and its associated complications (6, 7).

Since the initial identification of small ubiquitin-like modifier (SUMO) proteins in 1996, these proteins have increasingly been recognized as versatile and important post-translation modifications (PTMs) (8). SUMO proteins constitute a family of proteins that modulate their function through the processes of associating with other proteins (SUMOylation) and dissociating from them (deSUMOylation) (9). The modification of the substrate protein is reversible. Similar to ubiquitination, SUMOylation plays a critical role in regulating various cellular processes by modulating distinct substrates. This modulation influences the stability and subcellular localization of proteins, as well as impacting essential processes such as cell cycle regulation (10), DNA synthesis and repair (11), signal transduction, and cellular immunity (12).

## 2 SUMOylation

### 2.1 Overview of SUMOylation

The attachment of SUMO proteins to a cysteine residue within a protein is termed SUMOylation. In contrast, the removal of SUMO proteins from the substrate is referred to as deSUMOylation. *In vivo*, the SUMOylation and deSUMOylation processes reciprocally regulate the SUMOylated state of proteins.

The three-dimensional structure of SUMO proteins exhibits a similarity to that of ubiquitin, as both proteins belong to the conserved ubiquitin-like protein (UBL) family. SUMO proteins covalently bind to the lysine residues of target proteins through a cascade reaction facilitated by a series of enzymes. SUMO1, SUMO2, SUMO3, SUMO4 (13), and SUMO5 (14) are the five isoforms of SUMO proteins that have been described. Of these, there is widespread expression of SUMO1, SUMO2, and SUMO3 *in vivo*. The amino acid sequences of SUMO2 and SUMO3 are essentially identical but have only 46% homogeneity with SUMO1 (15–17). The expression of SUMO4 is predominantly observed in immune tissues, including the kidneys, spleen, and lymph nodes (18). Numerous studies have demonstrated a strong association between SUMO4 and the development of T1DM and T2DM (19–22). Additionally, SUMO5 is tissue-specific, highly conserved in primates, and is also involved in the formation and destruction of promyelocytic leukemia nuclear body (PML-NB) (14).

### 2.2 Mechanism of SUMOylation

The process of SUMOylation is analogous to ubiquitination, as the SUMO protein binds to the substrate protein through a series of enzymatic reactions. (**Table 1**) The principal enzymes involved in this process are, in order: the activation enzyme 1 (E1, Aos1/Uba2 heterodimer), conjugation enzyme 2 (E2, Ubc9), and SUMO ligation enzyme 3 (E3, such as PIAS, ZNF451, and RanBP2) (23–28). The PIAS family is the major SUMO E3 ligase and comprises PIAS1, PIAS2 (PIASx), PIAS3, and PIAS4 (PIASy) (29, 30). Subsequently, SUMO-specific proteases (SENPs) execute the deSUMOylation process.

**Table 1:** The enzymes of SUMOylation in mammal cells.

Enzyme	Homo sapiens	Mechanisms of enzymes in inflammation and diabetes	References
E1 activating enzymes	SAE1-SAE2 (Aos1/Uba2 heterodimer)	Overexpression of SAE1/Uba2 promotes glycolysis in cells, increases lactate secretion and enhances the expression of proinflammatory cytokines.	(133)
E2 conjugating enzymes	Ubc9	Both conditional ablation and overexpression of Ubc9 in pancreatic β cells impair β cell function and subsequently lead to diabetes.	(66)
E3 ligases	PIAS1, PIASx (2), PIAS3, PIASy (4)	PIAS-family proteins mediate the SUMOylation of Glis3, leading to decreased insulin transcription.	(134)
	ZNF451	ZNF451 inhibits the transcription of Smad3/4 and negatively regulates TGF-β signaling.	(135)
	RanBP2	Inhibition of the Insulin-like Growth Factor 1 Receptor (IGF1R)/RanBP2/SUMO1 complex formation restricts the expression of proinflammatory cytokines.	(136)
	STAT	SAE1/Uba2 promotes the phosphorylation and nuclear translocation of PKM2 through SUMOylation, thereby enhancing STAT5A expression and regulating glycolysis.	(133)
	Pc2	―	―
	MAPL	MAPL regulates inflammatory responses by suppressing NLRP3 inflammasome activity through SUMOylation.	(74)
deSUMOylation	SENPs 1–3 and 5–7	SENP2 modulates diabetes-associated lipid metabolism through regulating the deSUMOylation of SET domain bifurcated 1 (Setdb1).	(106)
		SENP6 and SENP7 regulate NLRP3 inflammasome activity through their deSUMOylation modification function.	(74)

**Figure 1:**
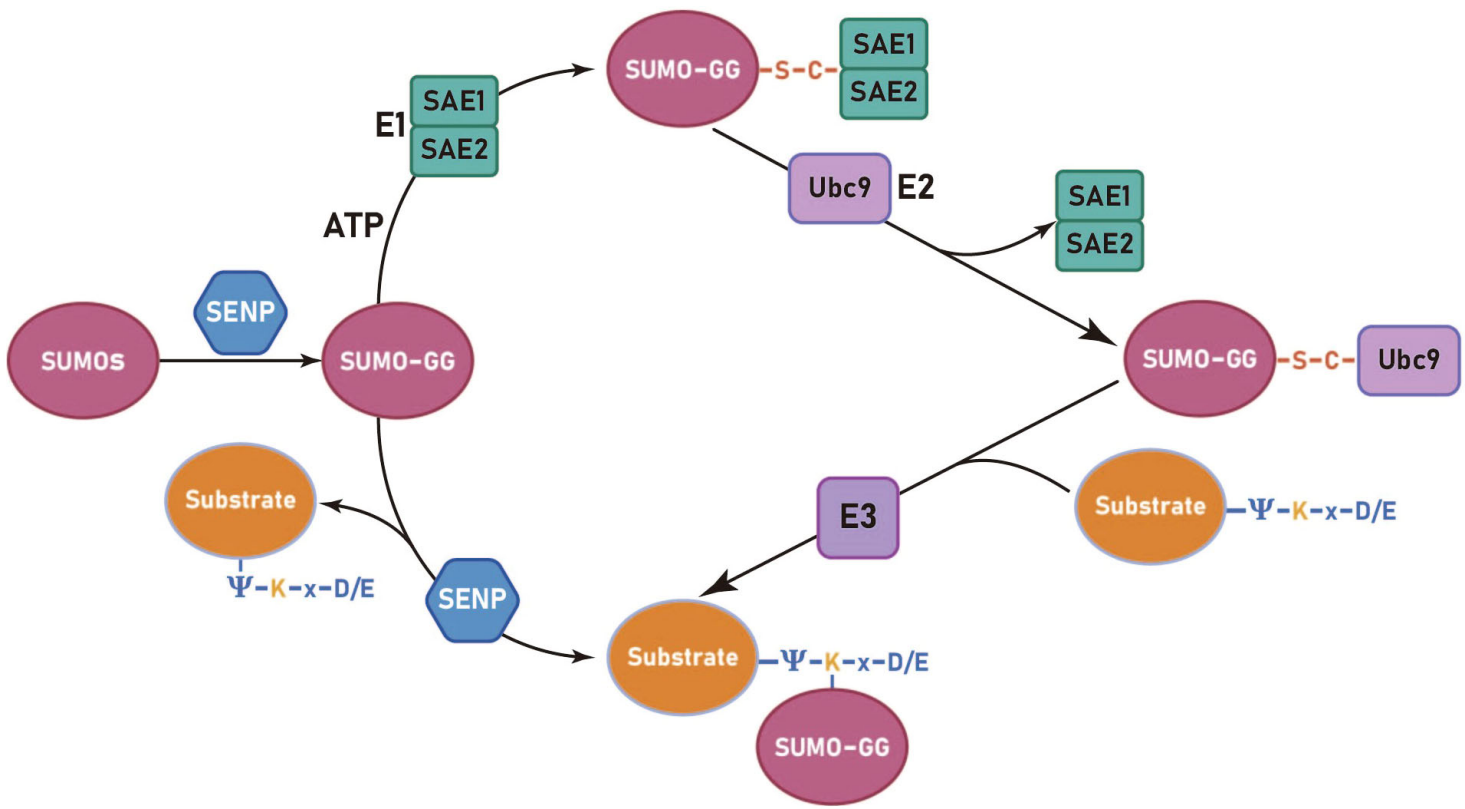
The SUMOylation cycle in mammalian cells involves several sequential steps. Initially, SUMO paralogues are cleaved by a SUMO-specific protease (SENP) to expose carboxy-terminal diglycine residues (GG). Subsequently, an ATP-dependent activation step is carried out by the activation enzyme 1 (SAE1 and SAE2), resulting in the formation of a high-energy thioester bond. The activated SUMO is then transferred from the E1 to the E2, forming a SUMO-E2 complex through a thioester linkage. Finally, the E2 enzyme, Ubc9, recognizes the conserved sequence Ψ-K-x-D/E (Ψ represents a hydrophobic group, K denotes lysine conjugated to SUMO, x signifies any amino acid, and D/E indicates an acidic amino acid, either aspartic acid or glutamic acid) on the substrate protein and facilitates the formation of an isopeptide bond between the SUMO and substrate protein, a reaction catalyzed by the E3 ligase.

The process of SUMOylation can be categorized into four principal stages: the maturation of SUMO, activation of SUMO by the E1 enzyme, the activated SUMO binds to the E2 enzyme and promotion of the conjugation of SUMO to the substrate by the E3 enzyme. (**Figure 1**) (і) The first step in SUMO maturation begins with SENPs degrading several amino acids at the C-terminus of SUMOs to expose the diglycine residues. (ii) Next, in the presence of ATP, the diglycine residues of mature SUMOs interact with the cysteine residue of the E1 enzyme, forming high-energy thioester bonds. E1s are constituted by two subunits: the SUMO-activating enzyme subunit 1 (SAE1 or Aos1) and the SUMO-activating enzyme subunit 2 (SAE2 or Uba2). Typically, these two subunits assemble into ATP-dependent heterodimers consisting of SAE1 and SAE2 (31, 32). The process of SUMO activation is accomplished through the mechanism above (33). (iii) Subsequently, SUMOs are transferred from the E1 to the E2, resulting in the formation of a SUMO-E2 complex. The only E2 binding enzyme identified is ubiquitin conjugating enzyme 9 (Ubc9) (34–36). (iv) Ultimately, under the catalytic influence of E3 ligases, Ubc9 directly recognizes the conserved sequence Ψ-Kx-D/E (Ψ represents a hydrophobic group, K denotes lysine conjugated to SUMO, x signifies any amino acid, and D/E indicates an acidic amino acid, either aspartic acid or glutamic acid) within the substrate. This recognition facilitates the conjugation of SUMO proteins to lysine residues of the substrate, forming isopeptide bonds and thereby completing the transfer of SUMO from the E2 enzyme to the substrate (37). The process of deSUMOylation is defined as the dissociation of SUMO from the substrate protein. The SENPs encompass various species, with seven members of the SENP family identified in the human genome, specifically SENPs 1–3 and 5–7 (38). In summary, SUMOylation is characterized by two major features: specificity and reversibility. The specificity is determined by the combined action of Ubc9 and E3 ligases, while the reversibility is achieved through the deSUMOylation process mediated by SENPs. This ensures the dynamic regulation of SUMOylation.

SUMOylation, a crucial post-translational modification of proteins, regulates key cellular processes through diverse molecular mechanisms. Notably, the fragile replication fork structure becomes particularly susceptible to chromosomal rearrangements and mutations during DNA replication. Studies have revealed that Pli1, an E3 SUMO ligase, mediates SUMOylation to perform dual functions: it not only maintains replication fork integrity but also promotes their translocation to nuclear pore complexes (NPCs) via poly-SUMOylation-induced SUMO chain formation. This regulatory mechanism orchestrates the precise repair of replication forks and is essential for preserving genomic stability (11).

Emerging evidence has highlighted the pivotal role of SUMOylation regulation in cell cycle control. Mechanistically, lactate has been shown to inhibit the deSUMOylation activity of SENP1 through zinc ion chelation at its active site, leading to enhanced APC4 SUMOylation. This post-translational modification triggers structural remodeling of the anaphase-promoting complex (APC/C), facilitating its interaction with UBE2C and thereby orchestrating proper cell cycle progression and mitotic regulation (10). Capitalizing on the essential role of SUMOylation in cell cycle regulation, scientists have developed TAK-981, a novel SUMO E1 inhibitor. This small molecule exhibits dual anti-tumor mechanisms: it not only arrests the cancer cell cycle through SUMOylation inhibition but also potentiates anti-tumor immunity by stimulating immune activation, with a specific emphasis on cellular immune responses. These pharmacological properties underscore its promising clinical applications in oncology (12).

## 3 Regulation of immune microenvironment by SUMOylation

### 3.1 Regulation of immune cells by SUMOylation

#### 3.1.1 SUMOylation and regulatory T cells

Regulation of immune homeostasis is well known to be the function of regulatory T (Treg) cells, which can exert immunosuppressive and inflammatory control effects by promoting the formation of anti-inflammatory (M2) macrophages (39), inhibiting T helper (Th) cell populations (40), and secreting inhibitory cytokines, such as interleukin-10 (IL-10) and IL-35 (41). SUMOylation is involved in maintaining peripheral T cell homeostasis and immune tolerance. Stimulation of the T-cell receptor (TCR) and cluster of differentiation 28 (CD28) can induce the production of reactive oxygen species (ROS), resulting in the accumulation of SENP3. SENP3 promotes the nuclear localization of BACH2 by mediating the deSUMOylation of the transcriptional repressor BACH2, thereby preserving the stability of Treg cells and their immunosuppressive function (42).

#### 3.1.2 SUMOylation and CD8+ T cells

TCR-induced ROS also prompts rapid translocation of SENP7 to the cytoplasm, thereby mediating the deSUMOylation, ubiquitination, and subsequent degradation of phosphatase and tensin homolog (PTEN) protein. In CD8 T cells, SENP7-dependent reduction of PTEN maintains metabolic fitness and effector function. Moreover, SENP7 enhances the activation of PI3K/mTOR signal transduction and is involved in the maintenance of glycolysis and oxidative phosphorylation (OXPHOS) in CD8 T cells (43). Several studies have indicated that SUMOylation can impede the presentation of major histocompatibility complex class I (MHC I) antigens, thereby promoting immune evasion by tumor cells. Conversely, the inhibition of SUMOylation has been shown to repair the MHC I antigen presentation mechanism, activate CD8+ T cells, and augment their cytotoxic efficacy against target cells (44).

SUMOylation is crucial for the growth of hepatocellular carcinoma (HCC) cells, and SUMOylation inhibitors TAK-981 and ML-792 effectively reduce SUMOylation in HCC cells. Furthermore, they enhance anti-tumor immunity by restoring the killing ability of T cells, promoting the activity of natural killer (NK) cells and inflammatory (M1) macrophages, activating innate immune cells, and modulating the intestinal microbiota (45). Moreover, the inhibitory effect of TAK-981 on SUMOylation in T cells has been demonstrated. The interferon (IFN)-like response mediated by TAK-981 inhibits the differentiation of Treg cells while enhancing various cytotoxic features of primary chronic lymphocytic leukemia (CLL)-derived CD8+ T cells, including degranulation (CD107a), and upregulated perforin, Granzyme B, and IFN-γ expression activity (46).

#### 3.1.3 SUMOylation and Th17 cell differentiation

Th cell subsets are important players in chronic inflammation and insulin resistance in DM, with the Th17 cell population garnering significant interest from researchers due to its heterogeneity (47, 48). Th17 cells represent a critical component of the pathogenic T cell population. A variety of parenteral autoimmune diseases and tissue inflammation have been linked to Th17 cells^48^. In the absence of SENP2, the co-transcriptional factor Smad4 serves as the modification site of SUMO1 to up-regulate the expression level and nuclear localization of Smad4 and promote the differentiation of T cells to pathogenic Th17 cells (49). PIAS4 catalyzes SUMO3 transcription factor retinoic acid-related orphan receptor gamma t (RORγt) to SUMOylation at the lysine residue 31 (K31), promoting the binding of two RORγt binding proteins (KAT2A and SRC1) to RORγt, which drives the differentiation and development of Th17 cells (50). Under the catalysis of two SUMO E3 ligases, PIASxβ and PIAS3, SUMO1 modifies the K54 of phospholipase C-γ1 (PLC-γ1) to regulate T cell activation and induce IL-2 production by facilitating the assembly of PLC-γ1 microclusters (51).

#### 3.1.4 SUMOylation and macrophage polarization

Macrophages are of significant importance in the regulation of the body’s immune response and metabolism. After polarization, macrophages form different subtypes: Th17 cells represent a critical component of the pathogenic T cell population. A variety of parenteral autoimmune diseases and tissue inflammation have been linked to Th17 cells, while M2 macrophages play an anti-inflammatory role (52). Studies have demonstrated that mice with conditional knockout of SENP3 exhibit diminished polarization of M1 macrophages and reduced production of pro-inflammatory cytokines under lipopolysaccharide (LPS) induction, thereby indicating that SENP3 plays an important pro-inflammatory role in LPS-induced lung injury and regulates M1 macrophage polarization by activating pyruvate kinase M2 (PKM2) in a hypoxia-inducible factor-1α(HIF-1α)dependent manner (53).

#### 3.1.5 Summary

SUMOylation exerts distinct effects on various types of immune cells, with regulatory mechanisms that differ across cell types and involve multiple aspects such as activation, differentiation and functional activities, affecting the secretion of downstream cytokines (such as IL-2, IL-6, IL-8, IL-10, etc.) or direct killing effects. While maintaining or breaking immune tolerance, SUMOylation also regulates the production and quantity of various inflammatory mediators, affecting the inflammatory response.

The above analysis reveals an antagonistic relationship between Treg cells and Th17 cells. SUMOylation has been demonstrated to regulate both of these cell types, suggesting that the modulation of Treg/Th17 cells under SUMOylation conditions may prove beneficial for the advancement and prognosis of autoimmune diseases. Additionally, the opposite effects on macrophage polarization and activated cells indicate that it is feasible to create an environment conducive to M2 macrophage polarization rather than M1 macrophage polarization by influencing SUMOylation, thereby controlling chronic inflammation or excessively amplified inflammatory responses.

### 3.2 SUMOylation and inflammatory response

#### 3.2.1 SUMOylation and the NF-KB signaling pathway

Post-translational modifications serve as critical regulators of immune signaling-related proteins. SUMOylated nuclear factor kappa B (NF-κB) p65 inhibits NF-κB activation and its downstream pathways in HCC cells by assembling with mesencephalic astrocyte-derived neurotrophic factor (MANF). As a transcription factor, NF-κB regulates gene expression activity, and its activation can result in the production of numerous inflammatory factors. The NF-κB signaling pathway is a classic pathway for regulating immune and inflammatory responses and is also key to the pathogenesis of DM and its complications. SUMO1 overexpression can also SUMOylate MANF and promote the nuclear import of MANF (54, 55).

Further study revealed that SUMOylated annexin-A1 (ANXA1) promotes inhibitory kappa B (IκB) kinase α (IKKα) degradation through selective autophagy, while numerous studies have indicated that IKKα/β-mediated phosphorylation-dependent degradation of IκB is the most critical step in canonical NF-κB signaling pathway activation, thereby inhibiting its overall activation (56).

**Figure 2:**
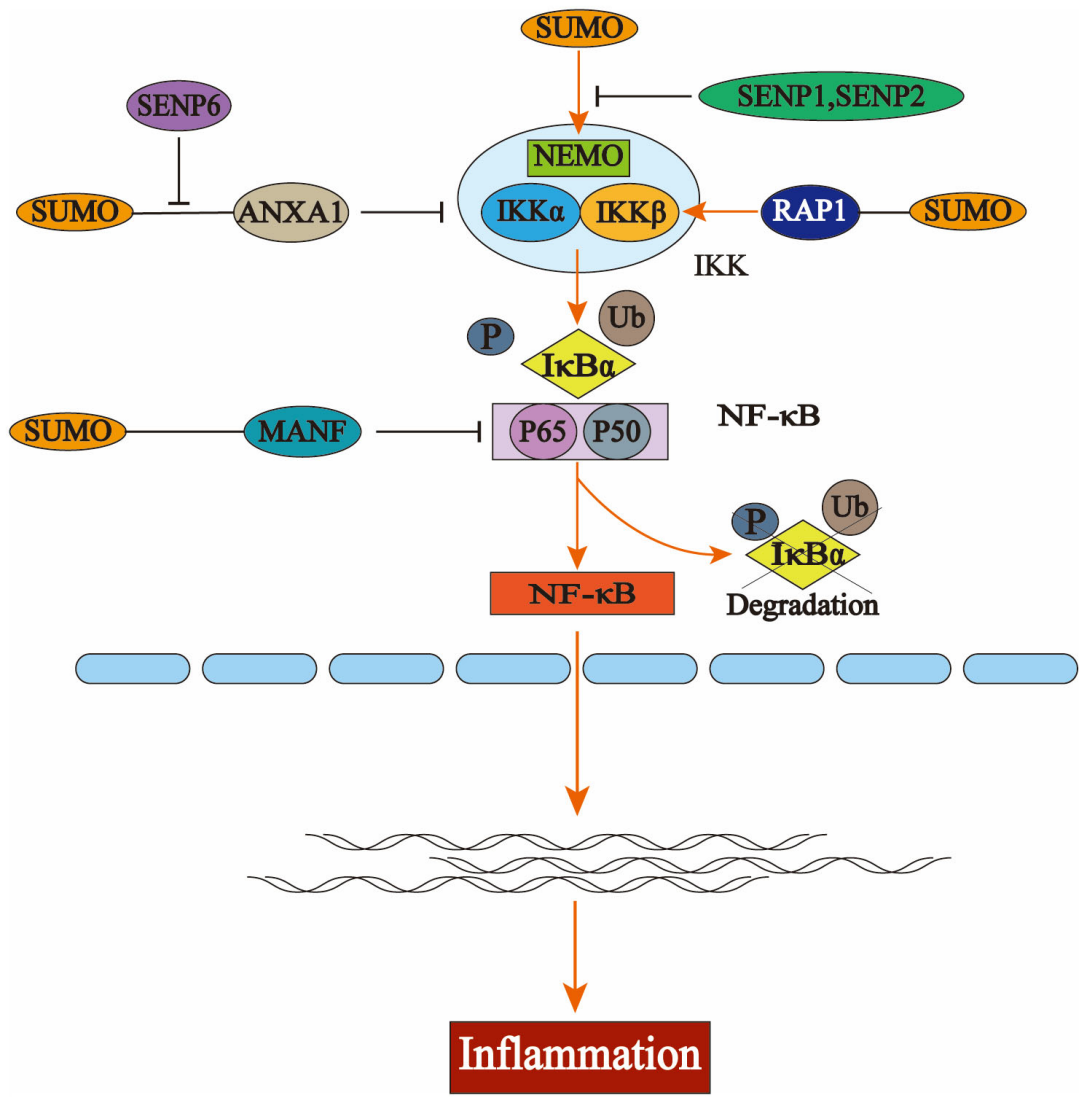
The activation process of nuclear factor kappa B (NF-κB) and the regulation of the NFκB signaling pathway by the small ubiquitin-like modifier (SUMO). In the resting state, NF-κB binds to the inhibitory protein IκBα to form an inactive trimer in the cytoplasm, thereby preventing its translocation into the nucleus. Upon stimulation by extracellular stressors, the inhibitory kappa Bn(IκB) kinase complex (IKK) is activated, resulting in phosphorylation and subsequent degradation of IκBα via the ubiquitin-proteasome system. This leads to dissociation of the trimer, allowing for nuclear translocation of the p50-p65 heterodimer that exhibits NF-κB activity and engages in gene transcription and protein synthesis. Several key components within the NF-κB pathway—including IκBα, NF-κB essential molecule (NEMO), P65, IKKα, and IKKβ—are subject to SUMO modification. The interaction between NEMO and SUMO facilitates the activation of IKK; similarly, SUMO-modified RAP1 enhances this activation process which ultimately promotes the degradation of IκBα and triggers NF-κB pathway activation. Additionally, the binding of annexin-A1 (ANXA1) or mesencephalic astrocyte-derived neurotrophic factor (MANF) to SUMO inhibits this pathway’s activation by interfering with key signaling molecules involved in NF-κB regulation.

Additionally, SENP6 could stabilize IKKα by deSUMOylating ANXA1, leading to inflammation (57). Under the regulation of the telomere-associated protein SLX4IP, the SUMO E3 ligase PIAS1 SUMOylates the telomere-binding protein RAP1, facilitating its interaction with IKK and subsequently leading to the activation of the transcription factor NF-κB (58). NF-κB essential molecule (NEMO) is a critical regulator in NF-κB. Intermittent hypoxia (IH) conditions enhance the SUMOylation of NEMO, resulting in NF-κB activation and upregulating the expression levels of tumor necrosis factor-alpha (TNF-α) and IL-6. SENP1-mediated deSUMOylation of NEMO can reduce the inflammatory response of microglia (59, 60). SENP2 can also inhibit the activation of the NF-κB signaling pathway through NEMO deSUMOylation, a mechanism that plays a crucial role in reducing doxorubicin resistance in breast cancer (61). The SUMO E3 ligase tripartite motifcontaining protein 60 (TRIM60) mediates the SUMOylation of TAK1 binding protein TAB2 at K329 and K562, and the SUMOylation of TAB2 interferes with the formation of TRAF6/TAB2/TAK1 complex to inhibit the MAPK/NF-κB pathway. This negatively regulates the innate immune response to toll-like receptor (TLR) signaling (62) (**Figure 2**).

#### 3.2.2 SUMOylation and the JNK signaling pathway

C-Jun-N-Terminal kinase (JNK) is a member of the mitogen-activated protein kinase (MAPK) family and serves as a critical component of the inflammatory signaling system. Under conditions of elevated H2O2, JNK can be activated as a substrate for SUMO1, with both molecules participating in the apoptotic death pathway caused by oxidative stress. Curcumin acts as an antiinflammatory antioxidant molecule to counteract this effect, which has important implications in retinal layer associated diseases (63, 64).

#### 3.2.3 SUMOylation and the NRF2 signaling pathway

Nuclear factor erythroid 2-related factor 2 (NRF2) has been demonstrated to be a key regulatory factor in maintaining redox homeostasis, typically existing in a low-activity state due to the negative regulation by kelch-like ECH-associated protein 1 (KEAP1), the substrate recognition component of the ubiquitin E3 ligase (65). Researchers have found that SUMOylation preserves and enhances the survival and function of β-cells while reducing the risk of DM. This process largely depends on the SUMOylation process of NRF2, a critical antioxidant factor, which plays a protective role in β-cells (66). SUMO2 has been demonstrated to modify NRF2 at K110 and K533, thereby influencing its transcriptional activity and effective nucleocytoplasmic localization (67). In addition, studies have shown that amyloid-β (aβ) can inhibit the binding of NRF2 and its activation signaling pathway by reducing the SUMOylation of both NRF2 and small musculoaponeurotic fibrosarcoma (sMaf) protein, which is essential for NRF2 activation. This process plays an important role in the onset and progression of Alzheimer’s disease (AD) (68). ROS could induce abnormal nucleolar activation of NRF2 through deSUMOylation of NRF2 mediated by SENP3 (69). Serine starvation diminishes SENP1 expression in HCC cells, facilitates SUMO1 modification of NRF2 at the conserved K110, enhances *de novo* serine synthesis, and perpetuates HCC tumorigenesis (70).

#### 3.2.4 SUMOylation and the inflammasome

The assembly of inflammasome is a critical factor in immune response, and SUMOylation is also involved in the activation of inflammasome as a post-translational modification mechanism. The non-obese diabetic (NOD)-like receptor (NLR) family pyrin domain-containing 3 (NLRP3) is essential for the activation of the NLRP3 inflammasome (71). The SUMO E3 ligase tripartite motifcontaining protein 28 (TRIM28) interacts with NLRP3, facilitating the SUMO1- and SUMO2/3catalyzed SUMOylation of NLRP3, thereby inhibiting its ubiquitination and subsequent proteasomal degradation. Consequently, TRIM28 stabilizes NLRP3 protein expression and enhances subsequent inflammasome activation (72). Other studies have similarly demonstrated that SUMO1 can mediate the SUMOylation of NLRP3 (73). In addition, previous studies have demonstrated that SUMOylation exerts an inhibitory effect on the activation of the NLRP3 inflammasome. For instance, the SUMOylation of NLRP3 mediated by mitochondrial‐anchored protein ligase (MAPL), identified as the first mitochondrial‐anchored SUMO E3 ligase, negatively regulates inflammasome formation, while SUMO-specific proteases SENP6 and SENP7 reverse this phenomenon (74). Additional experiments have also shown that the knockdown of SENP7 inhibits the activation of the NLRP3 inflammasome pathway (75). The nuclear zinc-finger and BTB domaincontaining protein 16 (ZBTB16) serves a positive regulatory role in the assembly of inflammasomes. The underlying mechanism involves ZBTB16 facilitating inflammasome assembly through the regulation of the adaptor protein ASC (apoptosis-associated speck-like protein containing a CARD) by SUMO1 modification at K21 and/or K109 (76) (**Table 2**).

**Table 2:** SUMOylation and inflammatory response.

Inflammatory responses	SUMO	Enzymes	Findings	References
JNK signaling pathway	SUMO1	―	They both participate in the apoptotic death pathway caused by oxidative stress.	(63, 64)
NRF2 signaling pathway	SUMO1	―	Serine starvation induces SUMO1 to modify NRF2 for self-*de novo* synthesis.	(70)
	―	Ubc9	Amyloid β (Aβ) inhibits the signaling of the endogenous antioxidant Nrf2 by decreasing its SUMO modification levels	(68)
	―	SENP3	ROS induces abnormal nucleolar activation of NRF2 through deSUMOylation.	(69)
	SUMO2	―	SUMO2 modifies NRF2 to affect its transcriptional activity and effective nucleocytoplasmic localization.	(67)
Inflammasomes	―	TRIM28	TRIM28 stabilizes NLRP3 protein expression and enhances subsequent inflammasome activation.	(72)
	SUMO1	―	SUMO1 mediates SUMOylation of NLRP3 protein.	(73)
	―	MAPL,SENP6, SENP7	SENP6 and SENP7 reverse MAPL negative regulation of inflammasome formation.	(74, 75)
	SUMO1	―	ZBTB16 promotes inflammasome by controlling the modification of the ASC.	(76)

## 4 Diabetes and immune system interactions

### 4.1 Overview of diabetes

Diabetes mellitus represents a group of metabolic disorders characterized primarily by chronic hyperglycemia, which serves as a key diagnostic criterion. In addition, patients with diabetes have typical clinical manifestations including polydipsia, polyphagia and polyuria. Diabetes-associated complications, categorized into macrovascular (primarily cardiovascular disease) and microvascular complications (including diabetic nephropathy, retinopathy, and neuropathy), can lead to multi-organ damage. These complications significantly impair patients’ quality of life while increasing morbidity and mortality rates (77). Diabetes is generally divided into four clinical categories, namely type 1 diabetes(T1DM), type 2 diabetes(T2DM), gestational diabetes (GDM) and other specific types of diabetes, and the vast majority of them are type II diabetes, accounting for 90%-95% of all diabetes (3, 78). This review primarily focuses on type 1 and type 2 diabetes, which represent the most clinically significant forms of the disease.

T1DM and T2DM exhibit distinct heterogeneity, with significant differences in their pathogenesis, clinical manifestations, and disease progression. T1DM predominantly affects younger populations, particularly children and adolescents, although its prevalence is highest in adults due to patient longevity (79). Autoimmune mediated beta cell destruction leading to insufficient insulin secretion is a recognized mechanism, symptoms include polydipsia, polyuria, weight loss, and in severe cases, diabetic ketoacidosis. Currently, the main treatment is insulin replacement therapy (80, 81).

In contrast, T2DM typically manifests later in life and is frequently associated with obesity. Its pathogenesis involves progressive insulin secretory dysfunction coupled with insulin resistance. Notably, patients with T2DM exhibit a significantly higher risk of cardiovascular events compared to their T1DM counterparts (82).

### 4.2 Interaction between diabetes and the immune system

#### 4.2.1 Interaction between T1DM and the immune system

It is well established that T1DM is an autoimmune disorder characterized by the destruction of β-cells mediated by T lymphocytes. T cells recognize islet antigens, drive islet autoimmunity and islet inflammatory infiltration, while activated autoreactive CD8+ T cells induce β-cell apoptosis through various mechanisms (83, 84), resulting in impaired insulin secretion. In addition to T cells, other immune components including innate immune cells (B cells, NK cells, macrophages, and neutrophils) and pro-inflammatory cytokines (such as TNF-α, IL-6, IFN-γ, and CXCL10) significantly contribute to disease progression by amplifying the autoimmune cascade (85–88).

Recent studies have demonstrated that β-cells are not merely passive ‘victims’ of autoimmunity, rather, they can also exacerbate the condition (89). Cellular alterations in β-cells, including enhanced immunogenicity, cellular senescence, oxidative stress, and endoplasmic reticulum dysfunction, can trigger the formation of novel antigenic epitopes, thereby amplifying immune responses. This self-perpetuating process represents a crucial mechanism through which β-cells contribute to T1DM pathogenesis (90). These results indicated that the islet - immune cell interaction promoted the progression of T1DM to a great extent, and the occurrence of T1DM could not be attributed to the immune system, and the involvement and metabolic changes of β cells were also worthy of consideration.

#### 4.2.2 Interaction between T2DM and the immune system

T2DM is acknowledged as a metabolic disorder characterized by insulin resistance and progressive insulin insufficiency, with obesity identified as the primary risk factor predisposing individuals to T2DM (91). Adipose tissue hyperplasia results in elevated free fatty acid (FFA) levels, triggering the release of monocyte chemokines and subsequent macrophage activation (92), while also facilitating macrophage polarization (93) that exacerbates the inflammatory response. Research has demonstrated that cytokines secreted by activated macrophages not only reduce adipocyte insulin sensitivity but also promote further macrophage infiltration and inflammatory signaling amplification. This cascade ultimately disrupts insulin receptor signaling pathways in adipocytes, resulting in systemic insulin resistance (94). Macrophages can also directly or indirectly cause β-cell dysfunction, leading to impaired insulin secretion (95, 96). In addition, other immune cells (B cells and T cells) and their secreted cytokines have also been shown to contribute to the development of insulin resistance and T2DM (97, 98).

T2DM is characterized by complex metabolic disorders, and in addition to obesity, the gut microbiome has also been shown to be involved in the development of T2DM (99). Disruption of the intestinal barrier leads to bacterial penetration, and recognition of bacterial components [mainly lipopolysaccharide (LPS)] by Toll-like receptors (TLRS) and their adaptor molecules leads to the activation of inflammasomes and inflammatory signaling pathways, resulting in insulin resistance and chronic inflammation (100). Furthermore, studies have demonstrated that IL-17 receptor deficiency under high-fat diet (HFD) conditions disrupts neutrophil migration in the intestinal mucosa. This impairment results in gut microbiota dysbiosis and subsequent LPS translocation to adipose tissue, ultimately contributing to insulin resistance and metabolic dysfunction. Additionally, IL-17 receptor deficiency impairs the phosphorylation and activation of key kinases in insulin signaling pathways, leading to compensatory hyperinsulinemia and exacerbation of insulin resistance (101).

### 4.3 Summary of the role of SUMOylation regulating the immune microenvironment in the development of diabetes mellitus

#### 4.3.1 The impact of SUMOylation on lipid metabolism

The function of adipose tissue is intricately linked to the pathogenesis of DM, with peripancreatic adipocytes (PAT) secreting pro-inflammatory cytokines that recruit immune cells, resulting in damage to pancreatic islet β-cells and exacerbating the progression of T1DM. The elevated SUMOylation of the component NEMO at the lysine-277/309 sites is pivotal in this process, as it induces NF-κB activation and cytokine expression in SENP1-deficient adipocytes. Moreover, NEMO SUMOylation and NF-κB activation in PAT exhibit greater sensitivity to variations in SENP1 expression and activity compared to other adipose depots (54). Relevant studies have also elaborated and summarized the role of SENP1-mediated protein SUMOylation in the pancreatic immune response, β-cell damage, and the progression of DM, while also proposing the use of NEMO inhibitory peptides or NF-κB inhibitors as potential therapeutic strategies for T1DM (102).

It is well established that inflammation plays a significant role in the pathogenesis of T2DM. In particular, inflammation within adipose tissue, resulting from both malnutrition and obesity, can impair insulin receptor function and diminish insulin sensitivity through the action of inflammatory mediators and immune cell infiltration (103). Recent evidence indicates that ubiquitin-conjugating enzyme E2 I (Ube2i) plays a crucial role in the maintenance of growth and normal function of white adipose tissue (WAT). Impaired WAT expansion, inflammatory responses, and adipocyte apoptosis were observed in Ube2i-deficient mice with adipocyte-specific deletion. These alterations led to metabolic abnormalities such as ectopic lipid deposition and insulin resistance in the liver, mirroring the insulin resistance seen in patients with adipose dystrophy. Notably, a sex difference was evident, with effects being more pronounced in females. This underscores the critical role of Ube2i in maintaining functional stability and normal expansion of adipose tissue, however, its specific downstream transcription factors and mechanisms of action require further investigation (104).

Another study demonstrated that adipocyte Ubc9 enhances the SUMOylation of Endoplasmic reticulum protein44 (ERp44) and promotes its covalent binding to endoplasmic reticulum oxidoreductase 1 (Ero1α), thereby exacerbating cell-to-cell transmission of endoplasmic reticulum stress (ER) signals and retention of Ero1α, which ultimately leads to disorders associated with ER stress. ER stress and impaired ER function disrupt systemic glucose homeostasis, resulting in abnormalities in lipid metabolism and insulin resistance. Ubc9 knockdown in adipocytes demonstrated that inhibition of SUMO modification attenuated high-fat-induced ER stress, facilitated lipolysis and energy metabolism, and restored ER function, ultimately leading to the alleviation of insulin resistance and obesity. These findings suggest that modulation of ERp44’s SUMOylation may represent a viable strategy for ameliorating obesity and insulin resistance in clinical settings (105). SUMOylation of SET structural domain bifurcation 1 (Setdb1) promotes Setdb1 to occupy the promoter site of the Pparg and Cebpa genes. Setdb1 functions as a histone methyltransferase responsible for H3K9 trimethylation, which increases H3K9 trimethylation in adipocytes and represses the expression of Pparg and Cebpa, ultimately leading to a reduction in lipid storage capacity. This process was validated in adipocyte-specific SENP2 knockout mice subjected to a diet rich in fats, which exhibited ectopic fat accumulation and insulin resistance pathology (106).

SUMOylation exerts diverse effects on lipid metabolism through its regulation of various target proteins. The primary mechanisms involve activation of adipose tissue inflammatory signaling pathways, induction of endoplasmic reticulum stress, and dysregulation of lipid homeostasis. Both adipose tissue inflammation and lipid metabolism dysregulation contribute to insulin receptor impairment, disrupted insulin signaling pathways, and subsequent development of insulin resistance and T2DM. Furthermore, adipose-derived inflammatory infiltration can induce pancreatic β-cell dysfunction, potentially exacerbating T1DM progression. These findings suggest that modulation of protein SUMOylation in adipocytes may represent a promising therapeutic strategy for mitigating insulin resistance and protecting β-cell function.

#### 4.3.2 The impact of SUMOylation on transcription factors and their downstream effectors

It has been reported that under high glucose conditions, retinal microvascular endothelial cells (mRMEC) exhibit significantly elevated levels of SUMO1 and SUMO2/3 proteins, along with enhanced stabilization of RUNX family transcription factor 1 (RUNX1) protein through SUMO2/3dependent SUMOylation, a process that is heavily reliant on the K182 and K144 residues of RUNX1. RUNX1 protein enhances the proliferation, migration, and angiogenesis of mRMECs, contributing to the exacerbation of diabetic retinopathy (DR) symptoms. This process can be alleviated by SENP1 overexpression, suggesting a potential correlation between SUMOylation and the severity of DR (107, 108). Similarly, high glucose induction can elevate the expression levels of SUMO1 and silent information regulator 1 (SIRT1) proteins in human lens epithelial cells (HLECs), moreover, SUMO1 overexpression enhances the SUMOylation of IκBα, thereby stabilizing this protein and facilitating its binding to NF-κB p65, which inhibits NF-κB p65 activation and oxidative stress. This mechanism protects the lens from high glucose-induced oxidative damage. Collectively, these findings suggest that SUMO1 may represent a novel therapeutic target for the treatment of diabetic cataracts (DC) (109).

NF-κB functions as a transcription factor that regulates gene expression activity, and its activation can result in the production of substantial amounts of inflammatory factors. It has been previously mentioned that several signaling molecules within the NF-κB pathway, including IKKα, NEMO, and p65, are subject to modification by SUMOylation. Contrary to previous reports indicating that high glucose and high osmolality diminish the interaction between IκBα and SUMO1, reduced SUMOylation of IκBα leads to decreased expression levels of IκBα, resulting in the activation of NF-κB signaling, which is implicated in the pathogenesis of diabetic nephropathy (DN). Therefore, novel therapeutic strategies targeting specific regulators of the NF-κB pathway may prove effective in treating DN (110).

Previous studies have also investigated the complex role of mouse SUMO2 (mSUMO2) in the development of autoimmune diabetes by establishing genetically modified mice with a enhanced repressor of NF-κB (IκBαΔN) and overexpression of mSUMO2, which demonstrated that Th1 cytokines were suppressed in the IκBαΔN T cells. Conversely, T cells with elevated levels of mSUMO2 showed a reduction in both Th1 and Th2 cytokine production. Furthermore, IκBαΔN completely prevented diabetes regardless of mSUMO2 overexpression, however, mice expressing only mSUMO2 exhibited susceptibility to diabetes comparable to that of wild-type mice. It is suggested that SUMO2 may differentially inhibit NF-κB expression at least in T cells, but the specific regulatory mechanism and the signaling molecules involved remain to be clearly elucidated (111). Similarly, the further development of DM and its complications through activation of the NF-κB pathway can also be observed through the enhanced phosphorylation and SUMOylation of IκB kinase γ (IKKγ) (30).

The NF-κB pathway plays a vital role in the development of DM through various mechanisms. For instance, deficiency of thioredoxin 2 (Trx2) in adipose tissue results in the stimulation of NFκB, which mediates the accumulation of the autophagy receptor p62, leading to mitochondrial autophagy and subsequently promotes insulin resistance and lipid metabolism disorders in T2DM, thereby linking NF-κB to metabolic disorders associated with insulin resistance (112). NF-κB also upregulates the expression of complement C3, thereby promoting the development of DM (113), C3 is a crucial component in the activation process of all three complement pathways and is not only associated with insulin resistance (114), reflecting the progression of metabolic derangement but also serves as a biomarker for T1DM and T2DM (115, 116). Epidemiological studies have demonstrated that elevated plasma levels of complement C3 can increase the risk of diabetic retinopathy, diabetic nephropathy, and neuropathy (113) in the general population (117). Furthermore, the pathogenesis of diabetes-related periodontitis mediated by C3 in patients with T2DM is supported by existing studies (118). Induced by hyperglycemia, the NF-κB signaling pathway mediates the activation of valvular interstitial cells (VICs), resulting in valvular calcification and dysfunction, which significantly contributes to the exacerbation of valvular calcification in patients with T2DM suffering from aortic stenosis (AS) (119).

Moreover, the NF-κB signaling pathway, along with its related inflammatory mediators, could represent a critical nexus in microRNA-92a-mediated diabetic cardiovascular disease (120). The aforementioned findings suggest that inhibition of the NF-κB signaling pathway may serve as an effective therapeutic target for DM. The mechanisms underlying NF-κB pathway activation are intricate and involve a diverse array of signaling molecules. It is anticipated that further investigation into potential sumo-chemical modifications regulating the NF-κB pathway will yield significant insights, particularly regarding their critical roles in the pathogenesis of TM and its associated complications.

The age-related decrease in c-Maf SUMOylation within CD4 T cells negatively correlates with IL-21 expression, which facilitates the differentiation of T follicular helper (Tfh) cells and the proliferation of granzyme B-producing effector/memory CD8+ T cells, thereby contributing to the development of autoimmune diabetes in NOD mice. Moreover, the findings indicate that SUMOdeficient c-Maf inhibits Death domain-associated protein 6 (Daxx)/Histone Deacetylase (HDAC) recruitment to the IL-21 promoter (IL-21p) and enhances histone acetylation mediated by CREBbinding protein (CBP) and p300. It is hypothesized that inhibitors of CBP/p300 may mitigate several effects induced by IL-21 (121). This study elucidates a potential link between the SUMO status of a single transcription factor and the pathogenesis of autoimmune diabetes, detailing the downstream signaling molecules involved (e.g., IL-21, Daxx, etc.) and their pathogenic effects supported by experimental data. It suggests that the low SUMOylation levels of c-Maf may serve as a precursor to autoimmune diabetes, thereby facilitating early preventive and therapeutic interventions. Additionally, the role and downstream effects of IL-21 offer novel insights for the treatment of T1DM.

The role of transcription factor c-Maf in the pathogenesis of autoimmune diabetes is further corroborated by reports indicating that c-Maf directly influences the expression of glucose transporter genes Sglt2 and Glut2. Additionally, c-Maf deficiency has been shown to ameliorate diabetic nephropathy associated with hyperglycemia and oxidative stress by downregulating Sglt2 and Glut2 expression (122).

IL-21, a cytokine primarily produced by Tfh, is an essential element of the immune system that modulates various immune subpopulations, including B cells and CD8+ T cells. It is instrumental in the pathogenesis of multiple autoimmune diseases, including DM (123). IL-21 has been demonstrated to exhibit pro-diabetic activity, contributing to β-cell destruction and the onset of spontaneous T1DM (124). Additionally, it is implicated in Tfh cell-mediated chronic vascular inflammatory responses and the progression of diabetic retinopathy (125). Clinical trials have demonstrated that the combination of anti-IL-21 antibodies and liraglutide preserves β-cell activity and enhances islet function, offering a promising approach to addressing the treatment of T1DM (126).

Besides IL-21, other cytokines, including members of the IL-12 family members (IL-12, IL23, IL-27 and IL-35) (127), along with IL-6 (128), TNF-α (129), have been closely associated with DM and its complications. Identifying these cytokines in relation to the development of diabetes mellitus also provides targeted therapeutic avenues for managing diabetes and its related conditions.

Similarly in NOD mouse CD4+ T cells, SUMO1 reduces IL-4 production by enhancing its interaction with c-Maf, thereby inhibiting c-Maf from binding to the IL-4p half-MARE site. This reduction is detrimental to the normal development of protective Th2 cells. Numerous studies have demonstrated that T1DM is associated with an attenuated Th2 response, and thus this pathway may be involved in the immune bias of T1DM (130).

SENP1 has been demonstrated to enhance cell death in the insulin secreting cell line (INS-1 832/13) and human islet cells, leading to islet secretory dysfunction that correlates with decreased expression of inducible nitric oxide synthase (iNOS) and nuclear translocation of NF-κB. In contrast, up-regulation of SUMO1 or knockdown of SENP1 can reduce the nuclear translocation of NF-κB and its target gene expression, suggesting that preservation of islet activity and restoration of islet survival can be achieved through the processes of SUMOylation and de-SUMOylation (131). Glycolipotoxicity and the pro-inflammatory cytokine TNF-α convert extracellular stress into pattern recognition receptor (PKR) activation via the second messenger ceramide and activated PKR interacts with the conjugating enzyme Ubc9 to promote SUMOylation-dependent stabilization of p53, whose enhanced transcriptional activity leads to cell cycle arrest and disrupts β-cell proliferation, thereby contributing to the progression of T2DM. These findings indicate that targeting the ceramide/PKR/Ubc9/p53 signaling pathway may represent an effective strategy for the treatment of T2DM (132) (**Table 3**).

**Table 3:** Molecular mechanism and pathogenesis of SUMOylation on diabetes mellitus and its complications.

Diseases	Molecular mechanisms	Pathogenesis	References
Diabetic retinopathy	SUMO2/3-dependent SUMOylation enhances RUNX1 protein stability.	RUNX1 protein promotes mRMEC proliferation, migration and angiogenesis.	(107, 108)
Diabetic cataract	IκBα SUMOylates and inhibits NF-κB p65 activation and oxidative stress.	Stabilization of IκBα protects the lens from high glucose-induced oxidative damage.	(109)
Diabetic nephropathy	SUMOylation of IκBα is reduced.	It is involved in the development of DN by activating the NF-κB signaling pathway.	(110)
	c-Maf deletion reduces the expression of glucose transport genes Sglt2 and Glut2.	It improves hyperglycemia and oxidative stress.	(122)
Autoimmune diabetes	SUMO2 differentially regulates NF-κB expression in T cells.	The role of SUMOylation in developing autoimmune diabetes *in vivo* remains to be investigated.	(111)
	Attenuation of c-Maf SUMOylation induces CD8 T cells to produce granzyme B.	Granzyme B causes β cell apoptosis.	(121)
T1DM	The enhanced SUMO1 conjugation to c-Maf resulted in decreased IL-4 production.	This is detrimental to the normal development of protective Th2 cells.	(130)
T2DM	PKR interacts with Ubc9 to promote SUMOylation-dependent stabilization of P53.	Enhanced transcriptional activity of P53 arrests the cell cycle of β-cell proliferation.	(132)

## 5 Conclusions and future perspectives

Recently, significant advancements in SUMOylation research has led to a deeper insight into the molecular mechanisms and enzymatic systems that are involved. More importantly, SUMOylation, as a novel reversible protein regulation process, plays a crucial role in the development of various physiopathological states within the body, offering new insights into the pathogenesis of multiple diseases. Building on this foundation, the present paper offers a comprehensive overview of how SUMO chemical modifications influence downstream signaling molecules by regulating the functions of proteins within the immune environment, thereby potentially impacting the pathogenesis of DM.

This review comprehensively examines the molecular mechanisms of SUMOylation and its regulatory effects on the immune system, with particular emphasis on their pivotal roles in the pathogenesis of both T1DM and T2DM. By exploring the intricate relationships among these components, we aim to clarify the critical function of SUMOylation in modulating the immune microenvironment and its implications for diabetes progression and associated complications.

Additionally, it summarizes several intricate molecular mechanisms, the majority of which are classical signaling pathways linked to immune homeostasis and inflammation, including adipose tissue inflammatory factors, NF-κB pathway activation, Tfh cytokines, and other cellular signaling pathways. This underscores the significant potential of SUMO in diabetes-related therapies. SUMOylation influences the activity of key protein kinases involved in the pathogenesis by disrupting downstream signaling pathways. Although several studies have supported this notion, most are based on similar animal models, and the detailed mechanisms of SUMO modification on the regulation of various physiological and pathological states of the organism remain inadequately elucidated. Furthermore, the proteins subject to this modification warrant further investigation. In future research, it is anticipated that a more comprehensive understanding of how SUMO-regulated key proteins induce islet inflammation and autoimmunity will be achieved, leading to the identification of new therapeutic targets for diabetes based on these key proteins and existing knowledge.
