# Corrigendum: Regulation of the immune microenvironment by SUMO in diabetes mellitus

**DOI:** 10.3389/fimmu.2025.1621597

**Published:** 2025-05-13

**Authors:** Yuting Zhuo, Shangui Fu, Yue Qiu

**Affiliations:** ^1^ Department of Endocrinology and Metabolism, Jiujiang Hospital of Traditional Chinese Medicine, Jiujiang, Jiangxi, China; ^2^ The Second School of Clinical Medicine, Jiangxi Medical College, Nanchang University, Nanchang, Jiangxi, China

**Keywords:** immune, SUMOylation, diabetes mellitus, inflammation, posttranslation modification, signaling pathway

In the published article, there was an error in affiliation(s) 1 and 2. Instead of “[^1^The Second School of Clinical Medicine, Jiangxi Medical College, Nanchang University, Nanchang, Jiangxi, China, ^2^Department of Endocrinology and Metabolism, Jiujiang Hospital of Traditional Chinese Medicine, Jiujiang, Jiangxi, China”, it should be “^1^Department of Endocrinology and Metabolism, Jiujiang Hospital of Traditional Chinese Medicine, Jiujiang, Jiangxi, China, ^2^The Second School of Clinical Medicine, Jiangxi Medical College, Nanchang University, Nanchang, Jiangxi, China”.

In the published article, there was an error regarding the affiliation(s) for “Yuting Zhuo”. As well as having affiliation(s) 1, they should also have “Department of Endocrinology and Metabolism, Jiujiang Hospital of Traditional Chinese Medicine, Jiujiang, Jiangxi, China”.

In the published article, there was an error in shared authors contribution list, author “Shangui Fu” has contributed equally to this work with author “Yuting Zhuo”. This has been corrected in the author list.

“Yuting Zhuo^1,2†^, Shangui Fu^2†^, Yue Qiu ^1*^”

†These authors have contributed equally to this work

The authors apologize for this error and state that this does not change the scientific conclusions of the article in any way. The original article has been updated.

